# Overexpression of CD44 accompanies acquired tamoxifen resistance in MCF7 cells and augments their sensitivity to the stromal factors, heregulin and hyaluronan

**DOI:** 10.1186/1471-2407-12-458

**Published:** 2012-10-06

**Authors:** Stephen Hiscox, Bedanta Baruha, Chris Smith, Rebecca Bellerby, Lindy Goddard, Nicola Jordan, Zaruhi Poghosyan, Robert I Nicholson, Peter Barrett-Lee, Julia Gee

**Affiliations:** 1 Welsh School of Pharmacy, Cardiff University, Wales, UK; 2 School of Biosciences, Cardiff University, Wales, UK; 3 School of Medicine, Cardiff University, Wales, UK; 4 Academic Breast Unit, Velindre Cancer Centre, Wales, UK

**Keywords:** Tamoxifen-resistance, CD44, erbB, Hyaluronan, Heregulin

## Abstract

**Background:**

Acquired resistance to endocrine therapy in breast cancer is a significant problem with relapse being associated with local and/or regional recurrence and frequent distant metastases. Breast cancer cell models reveal that endocrine resistance is accompanied by a gain in aggressive behaviour driven in part through altered growth factor receptor signalling, particularly involving erbB family receptors. Recently we identified that CD44, a transmembrane cell adhesion receptor known to interact with growth factor receptors, is upregulated in tamoxifen-resistant (TamR) MCF7 breast cancer cells. The purpose of this study was to explore the consequences of CD44 upregulation in an MCF7 cell model of acquired tamoxifen resistance, specifically with respect to the hypothesis that CD44 may influence erbB activity to promote an adverse phenotype.

**Methods:**

CD44 expression in MCF7 and TamR cells was assessed by RT-PCR, Western blotting and immunocytochemistry. Immunofluorescence and immunoprecipitation studies revealed CD44-erbB associations. TamR cells (Â± siRNA-mediated CD44 suppression) or MCF7 cells (Â± transfection with the CD44 gene) were treated with the CD44 ligand, hyaluronon (HA), or heregulin and their in vitro growth (MTT), migration (Boyden chamber and wound healing) and invasion (Matrigel transwell migration) determined. erbB signalling was assessed using Western blotting. The effect of HA on erbB family dimerisation in TamR cells was determined by immunoprecipitation in the presence or absence of CD44 siRNA.

**Results:**

TamR cells overexpressed CD44 where it was seen to associate with erbB2 at the cell surface. siRNA-mediated suppression of CD44 in TamR cells significantly attenuated their response to heregulin, inhibiting heregulin-induced cell migration and invasion. Furthermore, TamR cells exhibited enhanced sensitivity to HA, with HA treatment resulting in modulation of erbB dimerisation, ligand-independent activation of erbB2 and EGFR and induction of cell migration. Overexpression of CD44 in MCF7 cells, which lack endogenous CD44, generated an HA-sensitive phenotype, with HA-stimulation promoting erbB/EGFR activation and migration.

**Conclusions:**

These data suggest an important role for CD44 in the context of tamoxifen-resistance where it may augment cellular response to erbB ligands and HA, factors that are reported to be present within the tumour microenvironment in vivo. Thus CD44 may present an important determinant of breast cancer progression in the setting of endocrine resistance.

## Background

Widespread improvements in the treatment of estrogen receptor (ER)-positive breast cancer have been seen following the introduction of endocrine agents such as tamoxifen. Although recent clinical data point to the potential advantages of aromatase inhibitors over tamoxifen in some cases, tamoxifen remains a relevant and important intervention strategy, particularly for younger patients [1]. However, despite this, for a significant number of ER+ patients, their prognosis remains poor due to acquired resistance [2]. Whilst adjuvant endocrine therapy inhibits cell cycle progression in residual tumour cells, these agents are less effective at inducing cell death [3,4]. Thus chronic exposure of residual tumour cells to endocrine treatments may promote adaptive changes which ultimately facilitate development of a resistant phenotype [5]. In vitro, in vivo and clinical studies have provided an insight into mechanisms underlying acquisition of endocrine resistance and provide evidence for altered growth factor signalling, particularly involving erbB family members in this process [6].

An intimate relationship exists between the tumour and its surrounding microenvironment which can have a major bearing on tumour development and progression [7]. Importantly, our continued studies are suggestive that the endocrine resistant phenotype may be significantly sensitized towards factors likely to be present within the tumour microenvironment [8] which may also include growth factors that enhance erbB signalling.

We have recently identified that mRNA for the cell surface protein, CD44, is upregulated in endocrine-resistant breast cancer cells [9]. CD44, a family of transmembrane glycoproteins which arise through alternative splicing of a common gene [10], are able to modulate intracellular signalling through interaction with their extracellular matrix ligand, hylauronan (HA), and through formation of co-receptor complexes with various receptor tyrosine kinases [11-13]. Indeed, increasing evidence points to a role for CD44 as critical mediators of both growth factor- and HA-induced mitogenic and invasive signalling in cancer cells. For example, HA-mediated mitogenic signalling has been suggested to involve an interaction between CD44 and erbB family members [14,15] whilst the v6 isoform (‘CD44v6’) is critically required for HGF/SF-mediated c-Met activation in human cancer cells [11]. Clinically, several CD44 isoforms have been identified as potential metastatic determinants as a result of an association between their expression and extent of tumour spread and disease stage [16]. Interestingly, further studies have shown that CD44 can act to limit drug sensitivity in prostate cancer cells [17] and in clinical ovarian cancer [18] but a relationship between CD44 and endocrine response in breast cancer has not yet been reported.

The fact that the primary CD44 ligand, HA, is also frequently overexpressed in malignant cancers and has been shown to be an independent prognostic factor for overall survival in breast cancers [19] further suggests a role for CD44-mediated signalling in breast cancer progression.

In this study, we have investigated the hypothesis that upregulation of CD44 in an acquired tamoxifen-resistant state, may act to alter the phenotype of these cells through both modulation of erbB signalling and a sensitization of the cells to HA. We subsequently show here that both heregulin and HA promote migratory signalling in the presence of elevated CD44 expression through a process likely to involve modulation of erbB herterodimerisation. These data thus suggest that CD44 upregulation in vivo may have significant bearing on the progression of endocrine-resistant tumours through augmenting their cellular sensitivity to exogenous stromal factors. Importantly, our preliminary findings also suggest that the CD44v3 variant may act to limit endocrine response in the clinic. Taken together, these data suggest that CD44 may represent a novel point for intervention to suppress progression of endocrine-resistant breast cancer.

## Results

### CD44 is upregulated in tamoxifen-resistant MCF7 breast cancer cells

**Figure 1:**
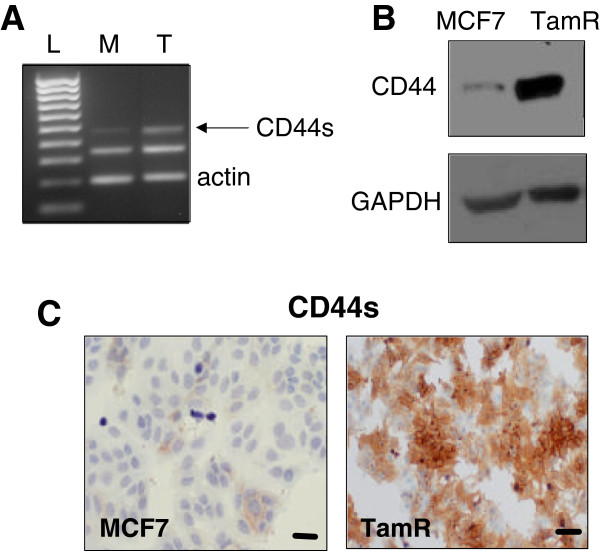
**CD44s is overexpressed in tamoxifen-resistant breast cancer cells.** (**A**) mRNA expression of CD44s was investigated through RT-PCR [L=DNA ladder; M=MCF7; T=TamR]. (**B**) Analysis of CD44s upregulation at the protein level in tamoxifen-resistant (TamR) cells was confirmed by Western blotting. (**C**) Immunohistochemical staining of TamR cells demonstrated significantly elevated levels of CD44 staining compared with their endocrine-sensitive counterparts (C, scale bar represents 20 Î¼m).

Our previous RT-PCR analysis of tamoxifen-resistant (‘TamR’) MCF7 cells revealed mRNA upregulation of the standard form of CD44 ([9] and confirmed in Figure 1A). Elevated levels of CD44 were subsequently confirmed at a protein level by Western blotting (Figure 1B), which revealed high levels of a protein of ~85kDa in TamR cells versus MCF7 counterparts corresponding to the expected size of CD44s. Analysis of CD44s expression using immunocytochemistry (Figure 1C) revealed intense staining in TamR cells, particularly at the cell surface.

### CD44 associates with the erbB2 receptor in TamR cells

**Figure 2:**
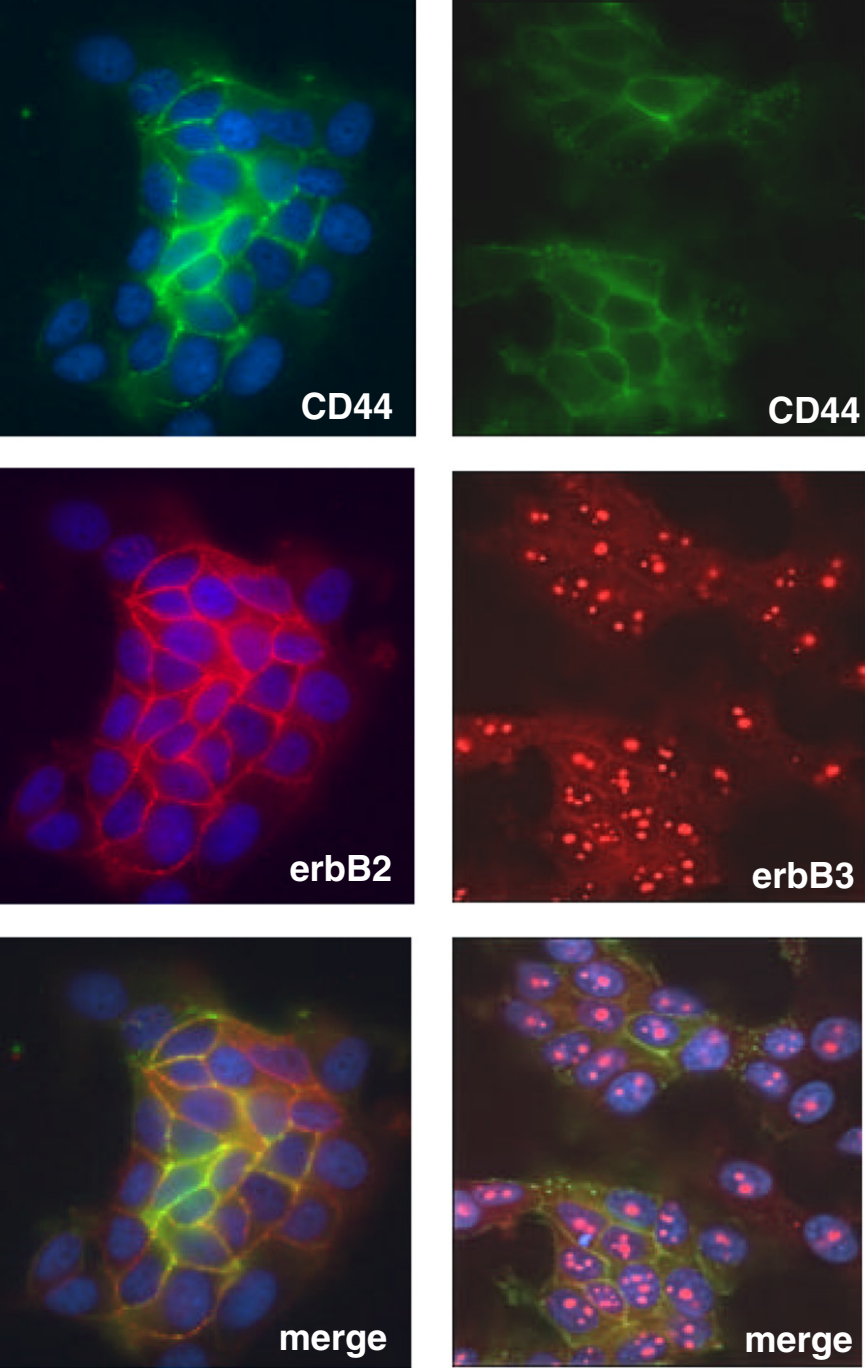
**CD44 is associated with erbB2 and erbB3 in TamR cells.** Immunofluorescence microscopy of CD44 (green) revealed a largely cell-surface location. Subsequent staining of erbB2 and erbB3 (red) and image overlay revealed apparent co-localisation (yellow/orange) of these receptors in TamR cells.

Signalling through the erbB family of receptor tyrosine kinases is known to play an important role in in vitro models of tamoxifen resistance and is mirrored in clinical disease [6,20]. Given the reported ability of CD44 molecules to modulate erbB receptor tyrosine kinase signalling though direct interaction with these proteins [13], we used dual immunofluorescence staining to investigate whether CD44s and erbB receptors were present in similar areas within the in TamR cell membrane. Staining of unstimulated TamR cells revealed an apparent co-localisation of CD44s and erbB2/erbB3 receptors (Figure 2). No endogenous association was observed between CD44s and EGFR (data not shown). These observations were additionally confirmed through immunoprecipitation. Interestingly, some of the erbB3 staining appeared to be located to the nucleolus as previously reported [21,22]. Immunofluorescent analysis of MCF7 cells was unable to demonstrate an association between CD44 and erbB receptors primarily as erbB receptor expression was barely detectable in these cells compared to their tamoxifen-resistant counterparts, as previously reported [6].

### Inhibition of CD44 expression suppresses endogenous erbB activity

**Figure 3:**
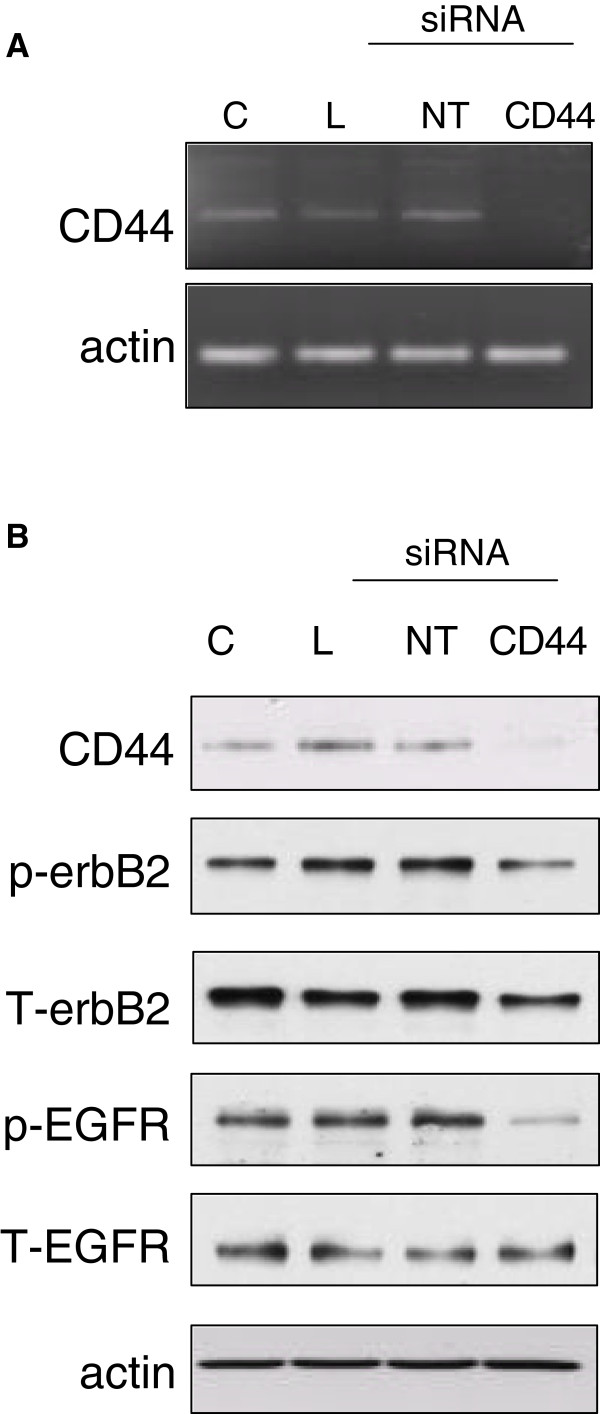
**siRNA-mediated suppression of CD44 reduces endogenous erbB activity in TamR cells.** Treatment of TamR cells with CD44 siRNA resulted in suppression of CD44 mRNA (**A**) and protein (**B**, upper panel). Treatment of TamR cells with siRNA delivery lipid (‘L) or a non-targeting siRNA (‘NT’) did not significantly affect CD44 expression compared to untreated control (‘C’) cells. In cells where CD44 expression was suppressed, the activity of EGFR was also reduced.

**Figure 4:**
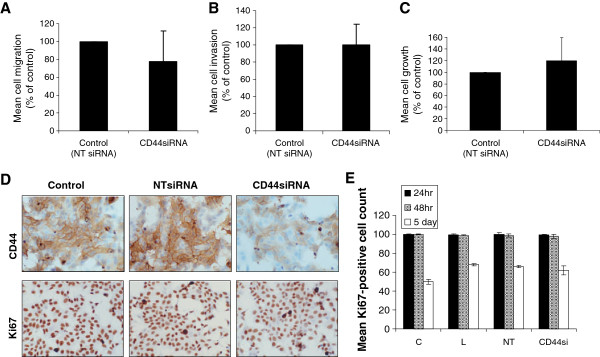
**Suppression of CD44 does not affect endogenous migration, invasion or growth of TamR cells.** Boyden-chamber based migration (**A**) and invasion (**B**) assays were used to determine the effects of CD44 suppression on the aggressive characteristics observed in TamR cells. Suppression of CD44 in TamR cells did not significantly alter either their migratory (A) or invasive (B) capacity. The growth of TamR cells after siRNA treatment was determined by MTT assay (**C**) and by staining for (**D**), and subsequent quantitation of (**E**), the proliferation marker, Ki67. Suppression of CD44 expression did not alter the growth characteristics of these cells.

We next explored the role of endogenous CD44 in TamR cells using an siRNA-based approach. Treatment of TamR cells with CD44 siRNA resulted in almost complete suppression of CD44 gene (Figure 3A) and protein (Figure 3B and Figure 4D) in contrast to the non-targeting (NT) siRNA control.

Given that CD44 was seen to co-localise with erbB2 in these cells and that CD44 has been implicated in erbB receptor activation [13], we investigated whether removal of CD44 would affect the endogenous activity of erbB2. Our data demonstrated that levels of endogenous phosphorylation of the EGFR were reduced following CD44 siRNA (Figure 3B). Although a modest suppression of erbB2 activity was also seen, this tended to be accompanied by a small reduction in erbB2 protein suggesting that basal levels of erbB2 activation are not greatly affected by the presence of CD44.

In light of these effects on intrinsic erbB activity, known to play an important role in TamR cell phenotype [6,23] we next explored the consequence of CD44 knockdown on TamR cell growth, migration and invasion. Suppression of CD44 in TamR cells resulted in a modest, but non-significant inhibition of TamR cell migration over fibronectin-coated membranes (Figure 4A) whilst invasion through Matrigel was not affected (Figure 4B). MTT growth assays revealed that TamR cells lacking CD44 displayed a similar rate of growth to that of their controls over a 5 day period (Figure 4C). Further quantitative analysis of Ki67 staining in CD44-deficient cells (Figure 4D and E) confirmed these data and showed no significant change in proliferative index versus their controls over this period.

### CD44 potentiates heregulin signalling in TamR cells

In view of our data supporting an association between CD44 and erbB receptors in TamR cells, we next addressed whether CD44 played a role as a regulator of ligand-induced erbB activity and signalling. For these experiments, CD44 expression was suppressed using siRNA prior to ligand challenge with heregulin Î²1 (Hrg), a potent activator of erbB signalling [24,25].

**Figure 5:**
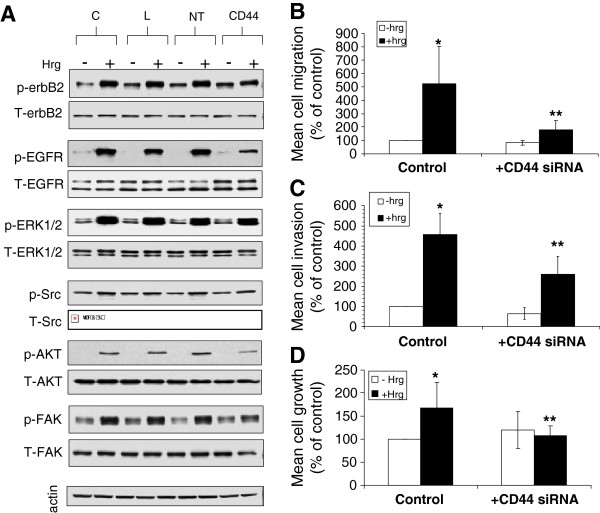
**CD44 suppression attenuates heregulin-induce invasive and migratory signalling in TamR cells.** TamR cells were treated with CD44 siRNA prior to treatment with the erbB lilgand, heregulin Î²1 (Hrg). Whilst Hrg promoted phosphorylation of erbB2 and the EGFR and associated downstream signalling pathways in control cells (**A**) with a resultant gain in TamR cell migration (**B**), invasion (**C**) and growth (**D**), this response was significantly attenuated in cells pre-treated with CD44siRNA. C: control (untreated) cells; L: siRNA transfection lipid alone; NT: non-targeting siRNA; CD44: CD44siRNA. Controls in figures B, C and D represent samples treated with NT siRNA. *p<0.05 vs. untreated control; **p<0.01 vs. Hrg-treated control.

Treatment of control cells (TamR cells treated with non-targeting siRNA) with Hrg resulted in phosphorylation of EGFR and erbB2 and activation of common downstream signalling components ERK1/2, Akt, Src and FAK (Figure 5A). However, in cells lacking CD44, response to Hrg was significantly attenuated (Figure 5A), particularly in the case of Hrg-induced EGFR phosphorylation. Whilst a modest inhibitory effect was seen regarding Hrg-induced ERK1/2 and Src activity, Hrg-induced AKT phosphorylation was largely suppressed following CD44siRNA treatment. Additionally, knockdown of CD44 expression significantly suppressing Hrg-induced migration (Figure 5B), invasion (Figure 5C) and growth (Figure 5D) in these cells.

### Hyaluronan induces migration in tamoxifen-resistant cells which overexpress CD44

Hyaluronan (HA) is the natural ligand for the CD44 receptor and promotes signalling events which are reported to mediate growth, survival, and migration in cancer cells [26]. As such, we wished to test the hypothesis that TamR cells, in which CD44 was over-expressed, would display a greater sensitivity towards HA versus their endocrine-sensitive MCF7 counterparts.

**Figure 6:**
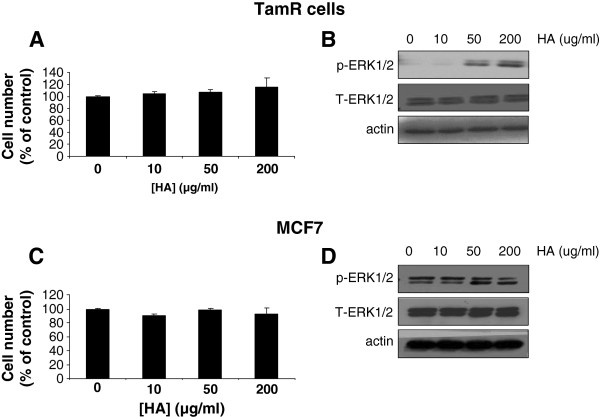
**Hyaluronan promotes growth, migration and invasion in TamR cells.** Treatment of TamR cells which naturally overexpress CD44 with the CD44 ligand, HA, resulted in a small but non-significant gain in growth (**A**) and an accompanying activation of ERK1/2 (**B**) which was not seen in MCF7 cells (**C**, **D**). Treatment of TamR cells with HA also enhanced their migratory capacity, an effect not seen in MCF7 cells (**E**; p=0.05 versus untreated cells). siRNA-mediated inhibition of CD44 expression in TamR cells prevented HA-induced cell migration (F, p=0.05 versus CD44siRNA-treated cells); accompanying images are immunohistochemical staining of CD44 in NT- and CD44siRNA-treated TamR wound assays.

Treatment of both cell types with increasing concentrations of HA (0-200g/ml) promoted a modest (but non-significant) increase in proliferation in TamR cells only (Figure 6A) which also corresponded to an small induction of ERK1/2 activity (Figure 6B). No such changes were observed in MCF7 cells (Figure 6C,D). Treatment of both cell lines with HA (200Î¼g/ml) significantly enhanced the migratory capacity of TamR cells only in in vitro wounding assays (Figure 6E), effects which were not apparent in the absence of CD44 (Figure 6F).

### HA-induced TamR cell migration involves erbB activation

**Figure 7:**
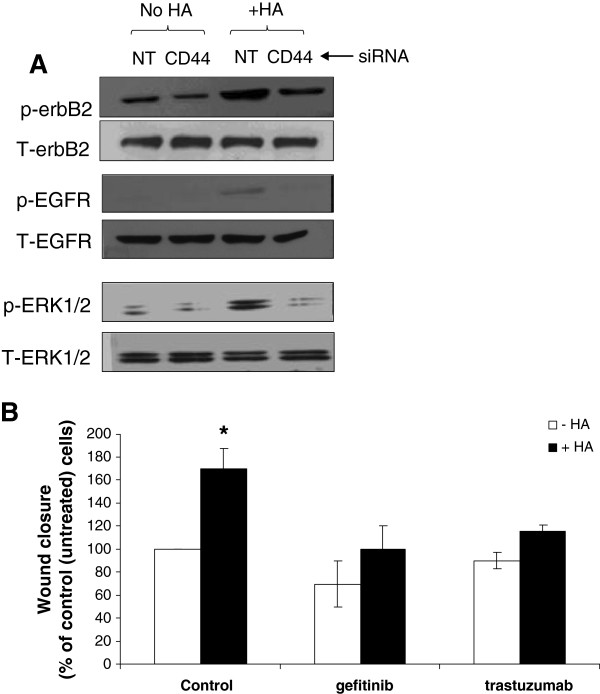
**HA-induced erbB receptor activation in TamR cells promotes cell migration.** (**A**) Treatment of TamR cells with HA is accompanied by upregulation of activated EGFR, erbB2 and ERK1/2; this was not apparent in cells in which CD44 expression was suppressed using siRNA. (**B**) To investigate the contribution of erbB signalling to HA-mediated cell migration, TamR wounding assays were performed in the presence of HA and in the presence or absence of the erbB2 inhibitor (trastuzumab) or the EGFR inhibitor (gefitinib). Inhibition of either erbB2 or the EGFR resulted in partial inhibition of HA-mediated TamR migration. *p=0.05 versus control.

To explore whether erbB activation was involved in HA-induced TamR cell migration, Western blotting was performed on untreated and HA-stimulated TamR cells in the absence or presence of CD44. HA induced EGFR, erbB2 and ERK1/2 activation in the presence of CD44 (i.e. cells treated with the non-targeting siRNA control) but not in cells treated with CD44siRNA (Figure 7A).

To further investigate the contribution of erbB signalling to HA-mediated cell migration, TamR wounding assays were performed in the presence of HA and in the presence or absence of the erbB2 inhibitor (trastuzumab) or the EGFR inhibitor (gefitinib). Inhibition of either erbB2 or the EGFR resulted in inhibition of HA-mediated TamR migration (Figure 7B).

### HA-mediated activation of CD44 alters erbB receptor dimerisation patterns in TamR cells

The ability of HA to promote erbB activation in the absence of erbB ligand suggested that CD44 may act to promote erbB dimerisation. To investigate this, we performed immunoprecipitation using EGFR and erbB2 antibodies in the presence or absence of HA and also following CD44 knockdown.

**Figure 8:**
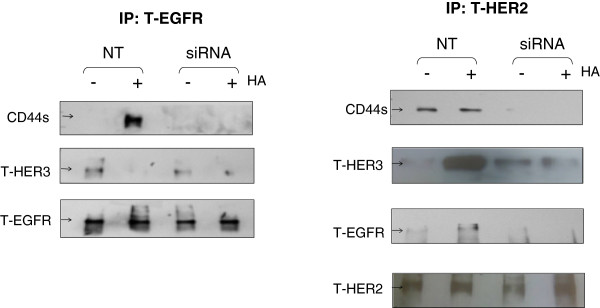
**HA treatment promotes erbB dimerisation in TamR cells.** TamR cells were treated with HA in the presence or absence of CD44siRNA and immunoprecipitation performed using EGFR, erbB2 or CD44 antibodies. Subsequent Western blotting revealed that HA treatment resulted in a shift in dimerisation pattern, with loss of EGFR:erbB3 and a gain in erbB2:erbB3, erbB2:EGFR and CD44:EGFR heterodimerisation. In the absence of CD44 (by siRNA), HA did not affect erbB dimerisation.

Analysis of endogenous erbB dimerisation patterns in TamR cells revealed presence of EGFR:erbB2, erbB2:erbB3 and erbB2:CD44 heterodimers whilst no signal was detectable for control samples immunoprecipitated with rabbit IgG (data not shown). No interaction was detected between EGFR and CD44 under basal conditions (Figure 8). HA stimulation of TamR cells resulted in a change in erbB heterodimerisation pattern, with apparent gains in erbB2:erbB3, erbB2:EGFR and EGFR:CD44 associations. In contrast, association between the EGFR and erbB3 were lost in response to HA (Figure 8). These changes in erbB dimerisation in response to HA were not detected in cells treated with CD44 siRNA save for erbB2:erbB3 heterodimers, which appeared to be partially reduced.

### Overexpression of CD44s in MCF7 cells enhances HA-induced erbB activation and a migratory phenotype

**Figure 9:**
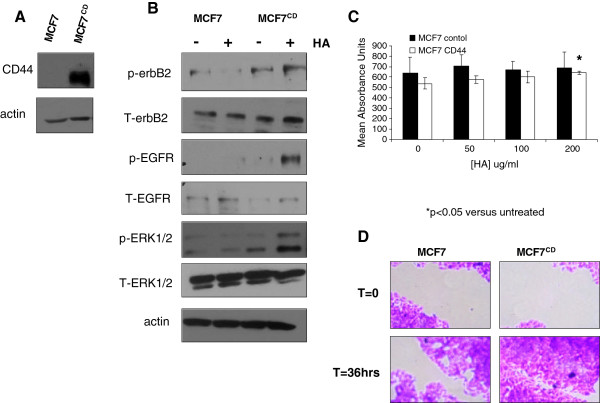
**Overexpression of CD44 in MCF7 cells augments their response to HA.** (**A**) The standard isoform of CD44 was transiently expressed in MCF7 cells and their response to HA subsequently examined using Western blotting and growth and migration assays. (**B**) HA induced EGFR, erbB2 and ERK1/2 activity to a much greater degree in CD44-expressing MCF7 cells than their non-expressing control counterparts. HA treatment of CD44-expressing MCF7 cells also promoted a small increase in proliferation (**C**) and a significant enhancement of their migratory capacity (**D**).

To further investigate the role of CD44 in breast cancer cells, we overexpressed CD44 in MCF7 cells which have barely-detectable endogenous levels of this protein (FiguresÂ 1 and 9A). Although overexpression of CD44s in these cells did not appear to significantly alter the endogenous activity of erbB2 or EGFR, treatment of these cells with the CD44 ligand, HA, resulted in phosphorylation of both the EGFR and erbB2 and downstream activation of ERK1/2 (Figure 9B), an effect not seen in MCF7 cells not over-expressing CD44.

The effect of HA on the proliferative and migratory capacity of MCF7 cells overexpressing CD44 was next tested. These experiments revealed that whilst HA did not significantly alter the growth characteristics of MCF7 control cells, MCF7 cells expressing CD44 displayed a partial response to HA in terms of growth (Figure 9C). Similarly, whilst HA did not induce migratory responses in control MCF7 cells, MCF7 cells expressing CD44 displayed a significant gain in migration in response to HA (Figure 9D).

### CD44v3 is associated with shortened outcome on tamoxifen in breast cancer patients

Our siRNA data implicates CD44 as an important mediator of erbB signalling in ER+ breast cancer cells with acquired tamoxifen resistance [27]. Previously, however, expression of CD44s has been shown to be a favourable prognostic marker in breast cancer. Since our siRNA approach suppresses a number of CD44 family members (C. Smith, unpublished observations) we wished to investigate the prognostic significance of additional CD44 family members. To investigate this further, we conducted preliminary analysis of CD44v3 expression in a small series (n=67) of ER+ clinical breast cancers by immunohistochemical staining. These data revealed that patients with >median CD44v3 expression (by H-score) had a shorter time to progression on tamoxifen (p=0.017) and a reduced overall survival (p=0.028) (Additional file 1: Figure S1).

## Discussion

The phenomenon of acquired endocrine resistance continues to pose a substantial hurdle to the effective treatment of breast cancer since clinical relapse frequently presents as metastases. Data from in vitro breast cancer cell models suggest that exposure to endocrine agents results in an induction of signalling pathways in the drug-responsive phase, which may include upregulation of growth factor receptor expression/activity, that can subsequently promote an endocrine-resistant state, both sustaining growth in the presence of endocrine agent and contributing to the development of an aggressive phenotype. Identification of such endocrine-induced elements, particularly those with a pro-invasive function, may represent potential therapeutic targets through which endocrine resistance might be circumvented and the resulting aggressive cellular characteristics associated with resistance suppressed. Such approaches have met with some success as evidenced through targeting of the EGFR [28] and Src kinase [29] in in vitro models.

In a previous study [9] we identified that expression of the CD44 gene was elevated in cell models of tamoxifen resistance. Although frequently used in conjunction with CD24 as a marker for cancer stem cells [30], CD44 proteins are also known to interact with, and modulate the activity of a diverse range of receptor tyrosine kinases including c-Met [31,32], VEGFR-2 [33], Her2 [34] and the EGFR [35], the latter two having been previously shown to limit endocrine response in ER-positive, endocrine-sensitive disease and contribute to a gain in invasive behaviour [6,20][36-38]. Given the reported interplay between CD44 and erbB receptors [15], we hypothesised that CD44 expression may represent a key element in the tamoxifen-resistant phenotype, potentially playing a role in the development of the adverse cellular characteristics accompanying this state.

Our data here revealed significant overexpression of the standard CD44 isoform (CD44s) at both gene and protein level in tamoxifen-resistant (‘TamR’) MCF7 cells compared to their endocrine-sensitive counterparts. However, CD44 did not appear to contribute to the intrinsic aggressiveness of these cells. This was surprising since CD44 suppression resulted in a reduction in endogenous EGFR activity and a modest suppression of erbB2 phosphorylation, receptors known to have a role as mediators of cellular growth and migratory responses. Interestingly, although we originally hypothesised that CD44 - erbB2 interactions might be dominant based on previous data supporting the interaction of these receptors in other cell types (e.g. [39]), our data here suggest that CD44:EGFR interactions are dominant in TamR cells; an observation further supported by our additional experiments which show a greater suppressive effect on EGFR activity versus erbB2 activity when CD44 is knocked down prior to stimulation of the cells with the erbB ligand, heregulin, or through hyaluronan stimulation.

Further analysis revealed that a number of common intercellular signalling components including ERK1/2, Src and AKT, known to be associated with erbB2 and EGFR-induced cellular responses, were not greatly affected on suppression of CD44 alone. These observations were further corroborated in MCF7 cells in which CD44 was overexpressed; whilst these cells demonstrated a small induction of endogenous EGFR and erbB2 activity, there was no significant change in signalling elements downstream of these receptors or to the cells’ intrinsic migratory nature. No effects on the endogenous growth of either cell type were seen following CD44 suppression nor did removal of CD44 either restore tamoxifen sensitivity to the TamR cells or modulate the response of MCF7 cells to tamoxifen (C. Smith, unpublished observations). Although together these data suggested a minimal contribution of CD44 to the basal phenotype of tamoxifen-resistant cells, further investigations revealed that CD44 expression significantly augmented the cellular response to the exogenous erbB ligand, heregulin, or to the CD44 ligand, hyaluronan, factors known to be present in the tumour microenvironment and which can influence tumour progression and spread. Indeed, breast cancer-associated fibroblasts, in contrast to those found in normal breast tissue, overexpress heregulin [40]; presence of this ligand promotes MMP9 expression and secretion in breast cancer [41] and is likely to enhance the cells’ invasive capacity in vivo. Although early investigations of heregulins in clinical breast cancer have failed to reveal an association between their expression and prognostic parameters [42,43], these studies tend to address expression of heregulin within the neoplastic epithelial cells rather than within the tumour stroma. Recently, however, a study analysing both epithelial and stromal heregulin expression in clinical tissues found that presence of heregulin within the stroma was significantly associated with disease recurrence and progression on endocrine therapy [44]. In light of our observations that CD44 augments heregulin sensitivity, we suggest that the presence of CD44 on breast tumour cells in clinical tissue may further aid identification of tumours less likely to respond to conventional therapy, an effect likely to be exacerbated in instances where tumour CD44 and stromal heregulins are co-expressed. Preliminary observations suggest that MCF7 cells which overexpress CD44s tended to display a reduced response to tamoxifen versus their wild-type counterparts, although this was not significant (S. Hiscox, Unpublished data). Given that overexpression of CD44 in MCF7 cells did not dramatically alter their intrinsic signalling capacity, one may hypothesize that the true consequence of CD44 overexpression would be revealed only in the context of the microenvironment, where exogenous ligands that can activate CD44 would be present. This hypothesis is supported by studies which have shown that CD44 expression can modulate chemotherapy response in breast cancer (e.g. [45]) although such reports have been primarily concerned with CD44, along with CD24, in the context of its role as a marker for intrinsically drug-resistant breast cancer stem cells. Others have demonstrated that high levels of CD44 are present on a doxorubicin-resistant MCF7-derived cell line [46]. However, in contrast, it has been recently described that high levels of CD44 in clinical breast cancer are associated with a more favourable prognosis [27].

It may also be likely that, rather than CD44 the level of stromal HA might represent a determinant of drug response and/or prognosis clinically and this remains to be fully elucidated. The tumour microenvironment is also a enriched with the CD44-ligand, HA, and in this context HA may have a significant bearing on breast cancer progression and spread through stimulation of CD44-mediated signalling. Binding of HA to CD44 is known to result in modification of the actin cytoskeleton system through activation of Rac1 signalling with resultant formation of membrane ruffles, cellular projections and induction of cell migration [47]. In addition, clustering of CD44, which may be induced by HA, has been suggested to sequester MMP9 at the cell surface [48] which may influence cellular invasion. Consistent with its role in promoting CD44-mediated migration and invasion, a number of studies have demonstrated that inhibition of HA production results in suppression of in vivo tumour development and metastasis [49-51]. In addition, HA expression in clinical breast cancer is a strong, independent, and negative predictor for patient survival [19,52]. Although HA-mediated activation of CD44-associated receptor tyrosine kinases, including the erbB2 receptor, has been described, little is reported on whether erbB dimerisation is involved in this process. Interestingly, our data supports a role for CD44 in mediating the effects of HA through re-organisation of erbB receptors, an event which may subsequently act to modify erbB signalling. Interestingly, HA appears to induce erbB2:erbB3 heterodimer formation in a CD44-dependent manner, a heterodimer pattern previously implicated in driving heregulin-induced gefitinib resistance [23]. That suppression of CD44 is able to deplete heregulin-induced activation of erbB signalling and also depletes HA-promoted erbB2:erbB3 association suggest a potential value of therapeutically targeting CD44 in breast cancer, potentially to improve the effect of current targeted therapies.

An important aspect of CD44 activity is the relative contribution of individual CD44 family members to the development of adverse cellular features. Although our evidence, along with published data, points to a role of CD44s in these events, it should be noted that our siRNA-based approach for suppressing CD44 likely results in knockdown of multiple CD44 isoforms including CD44v3 and CD44v6 (S. Hiscox and C. Smith, unpublished observations). Thus additional CD44 variants may also contribute to augmentation of heregulin of HA-mediated migratory signalling in TamR cells. This is particularly important given that some studies in the literature have failed to demonstrate a relationship between CD44s and tumour progression. For example, CD44v3 is protective against mammary tumour growth and metastasis in mice whereas CD44s is not [53]. In primary breast tumours, CD44v6 expression correlates with tumour size, nodal involvement and 5-year survival [54] and, whilst this study did not explore CD44s expression, similar studies comparing CD44s and v6 expression in invasive breast carcinoma have reported no correlation between CD44v6 and clinical outcome whereas CD44s appears to be a favourable prognostic factor [55]. Indeed, our own preliminary analysis of CD44v3 in a small series of breast cancers reveals an association with poor outcome on tamoxifen (Additional file 1: Figure S1). Thus, it may be that whilst some CD44 isoforms provide a protective role, others may promote adverse cellular features that could favour tumour progression. Moreover, although the HA-binding ability is common to all CD44 isoforms, the contribution of individual isoforms to erbB dimerisation and/or activation of these receptors is currently unknown. It is possible that one or more CD44 members may impart adverse cellular characteristics, potentially through activation of erbB or other receptors, onto tumour cells whilst other members may have a neutral or even tumour inhibitory role. Supporting such a hypothesis is data that suggests dominance of CD44s over variant CD44 isoforms with respect to promotion of tumour growth and spread. For example, whilst HA stimulation of CD44s is suggested to mediate breast cancer cell adhesion, motility and invasion, HA stimulation of CD44 variants regulate only cell motility [56]. Furthermore, induction of CD44s expression in MCF7 cells using a tetracycline-inducible system resulted in in vivo breast tumour invasion and metastasis to the liver [57]. Our in vitro data here suggests that overexpression of CD44s enhances breast cancer cell sensitivity to both erbB ligands and to HA and that subsequent stimulation of CD44-overexpressing cells results in modulation of erbB dimerisation and enhancement of migration. These observations may present a mechanistic explanation to recent reports which demonstrate that a shift from CD44 variant isoforms towards the standard isoform is required for cells to undergo EMT in vitro and in vivo [58]. Clinical studies appear to further establish CD44s over CD44 variants as a determinant of tumour progression with, for example, CD44s expression significantly correlating with lymph node involvement in lung adenocarcinoma metastasis in contrast to CD44v6 [59].

Our in vitro data here suggests that overexpression of CD44s enhances breast cancer cell sensitivity to both erbB ligands and to HA and that subsequent stimulation of CD44-overexpressing cells results in modulation of erbB dimerisation and enhancement of migration. These observations may present a mechanistic explanation to recent reports which demonstrate that a shift from CD44 variant isoforms towards the standard isoform is required for cells to undergo EMT in vitro and in vivo [58].

## Conclusion

Our data here show here that acquisition of tamoxifen resistance results in the overexpression of CD44 which augments the cells’ sensitivity to ligands commonly found within the tumour microenvironment. Our data thus support an emerging role for CD44 as an important determinant of breast cancer progression, particularly in the setting of endocrine resistance.

## Methods

### Cell lines and reagents

Tamoxifen-resistant (‘TamR’) MCF7 breast cancer cells were derived as described previously [6] and routinely cultured in ‘experimental medium’ (phenol-red free RPMI containing 5% charcoal-stripped, steroid-depleted foetal calf serum, 10IU/ml penicillin, 10Î¼g/ml streptomycin, 2.5 Î¼g/ml fungizone, 200mM glutamine), supplemented with 4-hydroxy tamoxifen (4-OH-Tam, 100nM). Endocrine-responsive MCF7 cells were routinely grown in phenol-red free RPMI containing 5% foetal calf serum and antibiotics as above. Experiments comparing MCF7 and TamR cells were carried out in experimental medium. Heregulin Î²-1 (Hrg, 10ng/ml) was purchased from Sigma Ltd (Dorset, UK), medium molecular weight hyaluronan (HA) from R+D systems (Oxford, UK) and CD44siRNA from Dharmacon Ltd (ThermoScientific, Leicester, UK). All antibodies were from Cell Signalling Technologies (New England Bioloabs, Hertfordshire, UK). All other reagents were from Sigma Ltd (Dorset, UK) and consumables from GIBCO (Paisley, Scotland) unless otherwise stated. Inhibitors were used at 1Î¼M (gefitinib) and 100nM (trastuzumab) unless otherwise stated.

### RT-PCR

MCF7 and TamR mRNA was reverse-transcribed to cDNA and amplified using primers F: 5’ GAC ACA TAT TGT TTC AAT GCT TCA GC 3’; R: 5’ GAT GCC AAG ATG ATC AGC CAT TCT GGA AT 3’ which correspond to the standard form of CD44 (CD44s). All PCR reactions included primers specific for Î²-actin as an internal control and were performed in a semi-quantitative manner using 27 cycles so that products were in the linear range of amplification. PCR products were visualised on a 1.0% agarose gel using ethidium bromide and photographed.

### siRNA-mediated suppression of CD44

siRNA knockdown of CD44 expression was performed using a pool of CD44-specific siRNAs (Dharmacon) according the manufacturers instructions. Briefly, TamR cells were incubated with transfection lipid alone (Dharmafect 1), lipid plus non-targeting siRNA (100nM) or lipid plus CD44 siRNA (100nM) in culture medium for 48 hours after which cells were harvested and used in the assays described below.

### Stable transfection of MCF7 cells with CD44 gene

A cDNA corresponding to the standard form of CD44 and inserted into the pSRÎ±-neo eukaryotic expression vector [60] was introduced into MCF7 cells as follows: briefly, MCF7 cells growing in log phase were washed twice in serum-free RPMI then incubated for 6 hours with serum-free RPMI containing the CD44 plasmid (50Î¼g) which had been pre-mixed with lipid (Lipofectamine 2000, Invitrogen) at 37Â°C and 5% CO2. The DNA/lipid-containing medium was then replaced with fresh G418 (400Î¼g/ml)-containing selection medium; cells were maintained in selection medium for a further 48 hours after which confirmation of CD44 expression was performed by Western blotting. Cells expressing high levels (equivalent to that of TamR cells) were selected and maintained in phenol-red free RPMI containing 5% foetal calf serum, antibiotics as above and G418 (200Î¼g/ml).

### Western blotting

Log phase cells (with or without prior treatment with CD44 siRNA and with or without stimulation using Hrg/HA as indicated in figure legends) were harvested, lysed in Western lysis buffer (1% Triton x-100 in 50 mM Tris, 150mM NaCl, 1mM EDTA, pH7.4 and containing protease/phosphatase inhibitors) and equal amounts of proteins resolved by 8% SDS-PAGE. Proteins were immobilised on nitrocellulose membranes and probed with CD44s (156-3C11, Neomarkers, Lab Vision, Cheshire UK) or phospho-specific antibodies against EGFR (pY845), erbB2 (pY1222), Akt (pS473), Src (pY419), FAK (pY861) and ERK1/2 (pT202/S204). Repeat immunoprobing was performed using pan antibodies and/or actin or GAPDH as a housekeeping gene. Representative blots are shown from a minimum of three separate experiments.

### Immunoprecipitation

Cells were lysed as described for Western blotting above and lysates containing 1 mg protein were immunoprecipitated using 1 Î¼g specific antibody (CD44s, erbB2 or EGFR) or mouse IgG as a control overnight at 4°C. Protein A agarose was added to the mixture, and the tubes were placed onto a rotary mixer at 4°C for a further 2 h. The immune complex was centrifuged at 3000 rpm at 4°C for 5 min and washed with ice-cold lysis buffer. After two further washings, the resultant pellet was resuspended in 20 Î¼l loading buffer containing DTT, boiled and processed as for Western blotting above.

### Immunocytochemical analysis of breast cancer cell lines

MCF7 and TamR cells were grown to log phase on tespa-coated coverslips. Cells were fixed with phenol/formal saline and expression of CD44s detected using antibodies as described above. Ki67 antigen detection was performed using a the MIB-1 antibody (Coulter Electronics, Luton, United Kingdom). Briefly, cells were treated as indicated then washed and fixed in formal saline. Primary antibody (MIB-1) was applied to the coverslips after blocking with PBS/Tween and incubation performed for 60 minutes. After washing in PBS, the secondary antibody (Mouse Envision, DAKO UK Ltd., Ely, Cambridgeshire) was applied to the coverslips for 75 minutes. After further PBS washing, the chromogen ("SigmaFast" DAB, Sigma, Poole, UK) was added to the cells for 10 minutes after which the coverslips were rinsed in distilled water. Samples were counterstained with 20% haematoxylin for 3 minutes and mounted for examination by light microscopy. Control coverslips (no primary antibody) were checked for non-specific binding before assessing staining intensity in the test samples. The percentage of Ki67-positive cells was estimated after counting at least 1000 tumour cells.

### Immunofluorescence

Log phase cells growing on coverslips were fixed in 3.7% formaldehyde prior to incubation with anti-CD44 and anti-erbB2 or erbB3 antibodies for 1 hour. After washing and incubation with FITC or TRITC-conjugated secondary antibodies (for CD44 and erbB2 respectively), coverslips were mounted and viewed with a fluorescent microscope. Images of CD44 and erbB2 were taken and merged to investigate co-localisation

### Migration/Invasion assay

Cells were seeded onto fibronectin-coated (100ug/ml, migration assay) or Matrigel-coated (5mg/ml, invasion assay), microporous membranes (8Î¼m pore size) at 50,000 cells/membrane and cultured for a period of 48hrs. Migratory cells were then fixed, stained and counted. Cell migration was quantified as the mean number of cells observed in each of 5 random fields of view per sample, in duplicate. Differences between groups were statistically compared using the student’s *t*-test.

### Wounding assay

Cells were grown to monolayer cultures in 24-well plates (in presence/absence of siRNA) and then ‘wounded’ by scratching the monolayer with a sterile yellow pipette tip. After gentle washing, medium was replaced with fresh medium containing appropriate treatments and the cells cultured for a further 36 hours. After this time, cells were fixed, stained with crystal violet and pictures taking using a bright-field microscope. Statistical analysis was performed using the student’s *t*-test.

### Proliferation assay

Cell proliferation was determined using the MTT assay. Briefly, cells were seeded into 96-well plates at 2000/well and left to adhere for 24hrs. After this time, medium was replaced with fresh medium containing treatments as described and the cells cultured for a further 5 days. The wells were then gently washed before addition of MTT solution (2.5mg/ml) for 4 hours after which the formazan crystals were solublized using 0.1% triton-X100 and the absorbance of the wells read at 590nm.

### Immunohistochemical staining for CD44v3 in breast cancer tissues

An exploratory series of breast cancers (n=67) [61] was used for immunohistochemical analysis of CD44v3. No patient had previously received any form of adjuvant endocrine or cytotoxic therapy. All breast cancer patients had received systemic anti-hormonal therapy and the duration of anti-hormonal response and survival from the initiation of therapy monitored for each patient by follow-up after surgery. Paraffin-embedded tissue sections were first subject to antigen retrieval by microwaving for 30 min in 0.1 M citrate buffer (pH 6.0). Following elimination of endogenous peroxidises with 3% hydrogen peroxide, immunostaining was performed using the CD44v3 antibody and subsequent incubation with DakoCytomation EnVision (30 minutes) and DAB (1:50 dilution). Negative controls were incubated with mouse isotype-sepcific control IgG (Dako).

All sections were assesses simultaneously by two observers blinded to the clinical data using a dual-viewing light microscope at x40 magnification. Matched control slides were checked for non-specific binding before assessment of staining intensity with a minimum of 2000 tumour cells evaluated per sample. The data were used to construct a CD44v3 histopathology score (H-Score, range 1-300) for each tumour specimen as detailed previously [62]. Immunohistochemical analysis, Mann–Whitney *U*-test was used to investigate the relationship between CD44v3 immunostaining and clinicopathological parameters. The Mantel-Cox (logrank) test and Univariate analysis (Kaplan-Meier method) were used to address the impact of CD44v3 on survival from initiation of therapy or on duration of therapeutic response. p<=0.05 was considered significant.

## Abbreviations

HA: Hayalurinic acid; erbB: Epidermal growth factor receotor family member; Hrg: Heregulin beta-1; ERK1/2: Mitogen activated protein kinase; EGFR: Epidermal growth factor receotor.

## Competing interests

The authors declare that they have no competing interests.

## Authors’ contributions

SH conceived the project, performed initial functional studies, drafted and revised the manuscript. BB and CS performed in vitro functional analysis, immunohistochemistry and immunoprecipitation. RB performed molecular transfection work. JG, PBL and RIN participated in the design of the study, study coordination and helped to draft the manuscript. NJ and LG contributed to gene and protein analysis studies; NJ and LF performed the clinical analysis. JG, RIN, SH, PBL and ZP provided intellectual input into interpretation of study data. ZP provided CD44 construct and assisted with transfection. All authors read and approved the final manuscript.

## Pre-publication history

The pre-publication history for this paper can be accessed here:

http://www.biomedcentral.com/1471-2407/12/458/prepub

## Supplementary Material

Additional file 1 **Figure S1.** CD44v3 is associated with poor endocrine response. Immunohistochemical analysis of CD44v3 expression in clinical breast cancers revealed an association between high (>median H-score) CD44v3 and both a shortened duration of response and overall survival. (PPT 92 kb) Click here for file
