# Supplementary material for: Overexpression of CD44 accompanies acquired tamoxifen resistance in MCF7 cells and augments their sensitivity to the stromal factors, heregulin and hyaluronan
Source: BMC Cancer. 2012 Oct 6;12:458. doi: 10.1186/1471-2407-12-458 (PMC3517483; doi:10.1186/1471-2407-12-458)
Supplement: Additional file 1 — Figure S1. CD44v3 is associated with poor endocrine response. Immunohistochemical analysis of CD44v3 expression in clinical breast cancers revealed an association between high (>median H-score) CD44v3 and both a shortened duration of response and overall survival. (PPT 92 kb) [file 1471-2407-12-458-S1.ppt]

## Slide 1
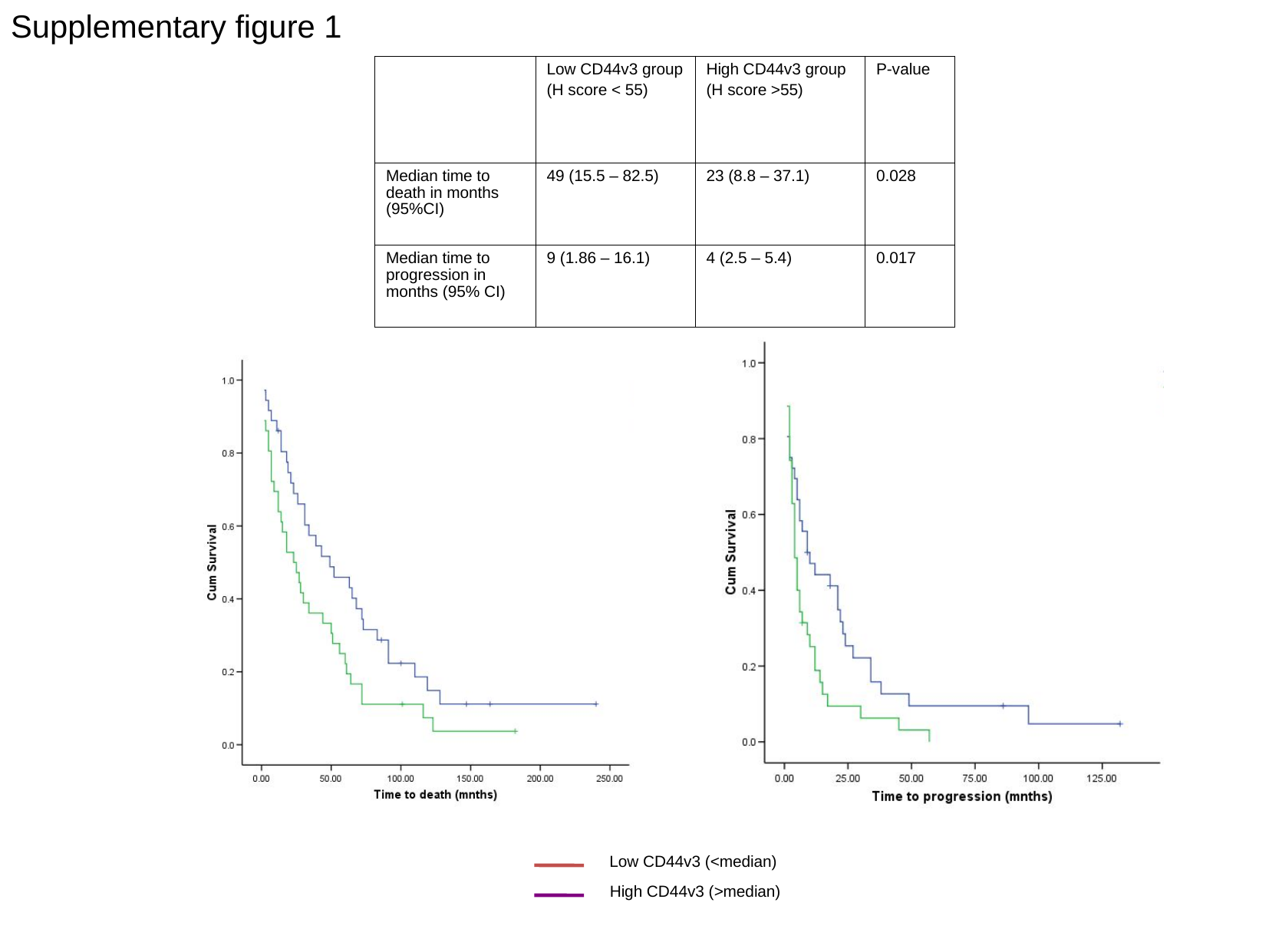

Supplementary figure 1
| | Low CD44v3 group (H score < 55) | High CD44v3 group (H score >55) | P-value |
| --- | --- | --- | --- |
| Median time to death in months (95%CI) | 49 (15.5 – 82.5) | 23 (8.8 – 37.1) | 0.028 |
| Median time to progression in months (95% CI) | 9 (1.86 – 16.1) | 4 (2.5 – 5.4) | 0.017 |
Low CD44v3 (<median)
High CD44v3 (>median)
